# Vulnerabilities among children of migrant rag pickers in UT Jammu and Kashmir: examining in the context of sustainable development goals 4 and 10

**DOI:** 10.3389/fsoc.2025.1695642

**Published:** 2026-02-04

**Authors:** Shabir Ahmad Najar, Wakar Amin Zargar, Bilal Ahmad Khan

**Affiliations:** Department of Social Work, University of Kashmir, Srinagar, India

**Keywords:** vulnerabilities, rag-pickers, children, education, phenomenological study

## Abstract

**Purpose:**

This study focuses on to scrutinize the experiences as well as the varying degrees of helplessness of children born into rag picking families. Within the agenda of Sustainable Development Goal 4 (Quality Education) and Goal 10 (Reducing Inequalities), this study demonstrates the systemic holes in learning and deepening cycles of dissimilarity slavery faced by children from migrant rag picking communities.

**Method:**

The study employed a qualitative, phenomenological approach. The research highlights the voices and perspectives of migrant rag picker about their children living in urban slums of Srinagar. These children may not take part in rag picking, but the coarse socio-economic and environmental conditions of the livelihood extremely affect them. The study is grounded on in-depth interviews, focused group discussions and observational data concerning the poor conditions, disenfranchisement, negligible schooling, compromised healthiness and chronic emotional abandon.

**Findings:**

Plentiful participants articulated a sense of disgrace, social isolation and persistent insecurity, often encountering unfairness not just in public spaces, but also in their own communities. Further, children of migrant rag pickers face various health issues and are not able to enroll their children in schools because of culture of poverty. Yet among these challenges, glimpses of hope, along with resilient aspirations infused with deep inner strength, persist.

**Conclusion:**

There is a pressing need for social policy that is both inclusive and intersectional, with a focus on providing educational access while eliminating structural socio-economic exclusion. The research advocates for a child-sensitive empathic approach to policymaking and social action by focusing on the children’s perspectives and offering a greater sense of humanity and respect while ensuring the policies will sustain equity and justice for all.

## 1 Introduction

Natural and cultural endowments characterize the union territory of Jammu and Kashmir. However, due to infrastructural stagnation, socio-economic turmoil, the region experiences iniquitous development. The region continues to suffer from backwardness (Owais et al., 2024). One of the most socio-economically disadvantaged groups in the region is the migrant rag pickers, entire families who make a living by harvesting, sorting and marketing city refuse. This activity, although illegal and perilous, is often the only employment option for migrants and local unemployed, as well as the economically disadvantaged people (Lal, 2022). As observed by Wani and Abid (2019) and Teotia and Kumar (2015), in Jammu and Kashmir, Srinagar, most migrant rag pickers originate from other parts of India, in search of the highly competitive informal job market in waste retrieval. This job primarily involves the daily retrieval of plastic, paper and other recyclable materials from the city’s streets and dumps (Teotia and Kumar, 2015; Ground Report, 2023). On a daily, they managed to fetch an average of 50 to 80 kilograms of recyclable materials, which they cart in rickshaws. The recyclables are then sold to scrap dealers for market rates of Rs 8/kg for plastic and 5/kg for cardboard etc. (Teotia and Kumar, 2015; Ground Report, 2023). The income of these families is modest; the money earned is not enough to support family.

Despite the fact that Srinagar produces 450–500 tons of waste daily, the municipal sanitation department only recognizes 4,000 workers as employees, completely overlooking the existence of rag pickers and their informal labour (Muzamil, 2021). These workers lack access to formal contracts and welfare support. The waste pickers also do not receive any medical care and suffer from many ailments, including, but not limited to infections, respiratory, musculoskeletal and cut-related issues (Teotia and Kumar, 2015; Ground Report, 2023). Following the 2019 abrogation of Article 370, many non-Kashmiri waste picker families chose to stay in Srinagar due to purported stable incomes and perceived community support (Wani and Abid, 2019; Ground Report, 2023). About 150 non-Kashmiri families opted to remain, while 300 others, out of an original population of 400 non-Kashmiri families, were living in makeshift shelters (Wani and Abid, 2019).

Children who are born into these families are denied the fundamental rights to a proper education, equality and a dignified life which perpetuates the cycles of poverty within these families. Families of migrant rag-pickers in Jammu and Kashmir are exposed to harsh socio-economic conditions (Gaon Connection, 2022). Children are particularly affected as they are exposed to a grim dual burden of inadequate socio-economic standing coupled with living in environments that are exploitative and riddled with filth. Most of these children are out of school or have dropped out due to poverty, lack of identification documents, and household responsibilities (Ahmed, 2024). The lack of evaluated policies and adaptive frameworks that address the needs of the children and inclusiveness fuel a further absence of education and greater poverty, and urban poverty among children from informal settlements and from nomadic families. Moreover, restricted availability of preschool and supportive services relevant to the specific age group neglects the development of a child’s intellect and social skills (Hassan, 2012).

SDG 4 (Quality Education) seeks to *“ensure inclusive and equitable quality education and promote lifelong learning opportunities for all,”* focusing on primary and secondary education for all, gender parity and equality in education and the education results achieved. For children belonging to rag-picking families, their goal in the economically disadvantaged region of Jammu and Kashmir requires tailored and nuanced strategies to address the context (UNESCO, 2021). Also, SDG 10 (Reduced Inequalities) urges everyone, no matter age, sex, ethnicity or economic standing to be empowered and have social, economic and political inclusion. Children born to families engaged in the informal economy, such as rag-pickers, are some of the people who are most often excluded from state benefits, institutional assistance, or opportunities which contravenes the core idea of welfare equity (UNDP, 2023). The inequality these children suffer is both social and structural, as it occurs within education, healthcare and the public and governmental systems which due to the lack of legal papers render them invisible and consequently, as a result of neglect, these children are excluded from fundamental measures of health and well-being, literacy and justice (Teotia and Kumar, 2015).

## 2 Methodology

This study utilized a qualitative research design framed within the phenomenological tradition. Specifically, Edmund Husserl’s transcendental phenomenology which explores the consciousness and human experience of individuals (Husserl, 1970). The aim was to reveal and understand the meaning structures surrounding the lived experiences of children of migrant rag pickers in the urban spaces of Srinagar, Jammu and Kashmir, through the lens of their primary caregivers. This design permits the researchers; in this case, to *“bracket”* prior assumptions and concentrate on the participants viewpoints that is phenomenology’s process of epoch (Moustakas, 1994). The study was conducted in certain areas of District Srinagar (i.e., Lal chowck, Batamaloo, Khanyar) which are noted for having a considerable concentration of migrating rag picker families. A purposive sampling strategy was used to identify 12 participants (which includes mothers/fathers or any other who member who acted as care taker of the children) from rag-picking families. Mothers/fathers were selected as primary participants because they are the primary caregivers and are the most responsible for their health, nutrition and education. To qualify, participants had to have resided in Srinagar for a minimum of 5 years, were actively engaged in rag-picking and had at least one child younger than 14 years.

To gather rich first-person experiences data collection adhered to phenomenological methods recommended by Creswell (2007) using in-depth semi-structured interviews and focus group discussions (FGDs). A total of 12 in-depth interviews and 4 FGDs were held. Data collection process continued till data saturation point was achieved. Thematic interview guides targeted child health, sanitation, school attendance, social exclusion and emotional well-being. The interviews were held in Urdu language, which was common to both the researchers and the participants. All interviews were recorded with the participant’s permission and subsequently transcribed and translated to English. Maintaining confidentiality and anonymity of participants was adhered to strictly in accordance with ethical research practices. During the course of the interviews, additional probes and follow-up questions were introduced whenever necessary to prompt participants to provide more detailed explanations and to ensure clarity in their responses. The modified Stevick-Colaizzi-Keen method of data analysis proposed by Moustakas (1994), using horizontalization, meaning clustering and creation of textural and structural descriptions of experiences was applied to the collected data. This method allowed the researchers to distill the essences of the experiences that participants shared.

To begin with, the researchers immersed themselves in the data by listening to and transcribing audio recorded interviews with rag pickers, observations from the field and focus group discussions (FGDs) alongside noting down field notes. In this early stage of familiarization, researchers recorded a variety of notes that captured powerful impressions such as the predominant health and educational issues surrounding waste work and geopolitics of migration, which were very critical in understanding patterns and layering meanings embedded in the data. In this phase it was also critical to develop systematic initial codes that capture the essence of constituent segments of data within them. For a very precise example, the statement made by a rag picker who said, “People call our children illiterates, dirty and avoid them,” was coded as educational backwardness. Other emerging descriptors also include: low wage, health risks and residential problems. The research team set out using NVivo and under Braun and Clarke (2006) guidelines, made sure all relevant data was captured coded inclusively through an inductive approach. Further, the researchers organized possible themes to capture broader structures using related codes. The themes were articulated and organized into more refined versions capturing their essence and importance.

To ensure the credibility and trustworthiness of the findings, the study used member checking in which some participants were invited to verify thematic interpretations. Trust was also achieved through member checking and was enhanced by triangulation in which cross-validation between interview narratives and field observations, as well as notes from FGDs was conducted. Also, through interviews, participants were non-structured observers of the physical and environmental conditions through a structured checklist. These narratives were contextualized through the observation of inadequate housing, sanitation and environmental risks. Such observations helped enrich the phenomenological descriptions and build a more complete image of vulnerability. This methodology is based on the premise that subjective human experience is sociologically important, particularly in relation to marginalized communities. Through this approach, the intent of the study was not only to document the suffering, but also to portray the experience, interpretation and narrative frameworks constructed by the sufferers in relation to systemic disregard, poverty and social exclusion.

## 3 Findings

### 3.1 Socio-demographic profile of rag pickers

The information gathered from 12 rag-picker families based in UT Jammu and Kashmir indicates a particularly marginalized socio-demographic profile (Table 1 as mentioned below). A sizable majority of families (75%) are nuclear, while a minority (25%) is joint. The population’s caste composition is largely dominated by Scheduled Castes account for (58%) of the population, with a small (42%) segment from Muslim backward classes. No adults in the sample reported being educated, which points towards the population’s persistent challenge of high illiteracy levels. This lack of education is also accompanied by greater child neglect complacency in health and hygiene understanding. Each family has significant number of family members, which contributes to the congested living conditions in makeshift shelters. Within the 12 families, a total of 23 children were recorded. Out of 23 children in the eligible school-going age (6–14 years), only 07 (30%) are actively attending school, while a majority of 16 (70%) are either un-enrolled or dropouts. The study also revealed that on an average, these families earn up to Rs. 5,000–7,000 per month. These statistics clearly indicate intergenerational illiteracy, limited educational opportunities, congested families and possible child neglect. The children under 8 years old seem to spend most of their time trapped in the filthy surroundings with no facilities for education or adult supervision. The data indicate that children, even under very difficult conditions, tend to go unsupported by formal structures. The lack of education possible coupled with the economic conditions of these families suggests that there are structural inequities which restrict children in these families from thriving. Such environments not only threaten children’s wellbeing but violate their fundamental rights and holistic development. These socio-demographic aspects are important in comprehending the lived vulnerabilities of the children of rag-pickers in the region.

**Table 1:** Socio-demographic profile of the participants.

Theme	Class	Response	Percentage
Family type	Joint	03	25
	Nuclear	09	75
Caste/religion	Scheduled caste	07	58
	Scheduled tribe	00	00
	Muslim minority	05	42
Educational status of parents	Literates	00	00
	Illiterates	12	100
Family size	2–3 members	01	8
	4–6 members	04	33
	7–8 members	05	42
	9 and above	02	17
Children going school (out of 23)	Yes	07	30
	No	16	70
Total number of participants	*N* = 12		

### 3.2 The living conditions of children in temporary settlements

This part analyzes the singular and collective day-to-day experiences of rag-picker’s children living within makeshift settlements in the Union Territory of Jammu and Kashmir. Children in these temporary settlements face multiple forms of structural and environmental violence. Such ephemeral settlements are often situated on the peripheries of the city or in close proximity to dumpsites. In addition to being the epicenter of social isolation, children are bound to face multiple health complications. Most of these families reside in shelters made of discarded plastic sheets, tarpaulin and other scraps that they have collected from the nearby dumps. These shelters do not shield children from the harsh cold of winter or the suffocating heat of summer. The settlements lack basic hygiene services like clean drinking water, sanitation and electricity. This lack of sanitation services makes children and families defecate in the open, vulnerable to infections and diseases. Many children living close to stagnant water pools are further exposed to mosquito and water-borne diseases.

The settlements experience the lowest standards of public infrastructure. The living conditions are filthy because there are piles of garbage everywhere which brings animals and insects. The children have no clue of the health risks and dangers involved as they spend time playing near the garbage and waste. Not only this, but many children are seen barefoot walking on extremely sharp shards of broken glass, rusted metal and filthy waste. Most families do not have legal identification documents which prohibit them from accessing housing or programmes on resettlement. One participant narrated that:

*I found always my children not able to sleep properly due to lack of proper heaters or bedding to stay cozy and warm. Winters affects the health of my children badly, as my home is not concrete and lacks all facilities. During winter my children remain ill due to severe cold” (Radika).*

These families, despite their difficulties, are seldom included or considered in discussions regarding urban development and are invisible in the municipal documents. Children lack access to basic necessities. These children and their families have almost no chance of accessing the services and resources they require to break free from the persistent cycles of poverty and social isolation.

*“Another participant mentioned that the lack of proper clean water sources forces them to fetch water from broken public pipes and nearby filthy drains. I am not rich and cannot afford to purchase water bottles for my children. I am leaving in tent and here I do not have any water facility. My children are living a miserable life and unfortunately I am not able to do anything for my children. My occupation is not helping me to generate enough money” (Kamal).*

It can interpreted from the above narratives that migrant rag pickers children are prone to various problems and are living under hazardous conditions that impacts their overall development.

### 3.3 Vulnerabilities of the children of rag pickers

This segment highlights the primary vulnerabilities of the children of rag-pickers in Union Territory Jammu and Kashmir with regard to four pressing concerns of SDG. This study analyzes the impacts of systemic neglect of marginalized socio-economic groups, health, education and social *“invisibility”* on children of rag-pickers. The Table 2 below illustrates the realities which have been considered in connection with the certain global development goals. Throughout the findings, pseudonyms are used to maintain the confidentiality and dignity of the participants.

**Table 2:** Field realities concerning vulnerabilities of rag pickers children against selected SDG 4 and SDG 10 targets.

Targets	Description	Field reality
4.1	Ensure that all girls and boys complete free, equitable and quality primary and secondary education.	Frequent dropouts due to work pressure.
		Lack of school enrollment. Discrimination by peers and teachers.
		Irregular attendance.
4.2	Ensure that all girls and boys have access to quality early childhood development, care and pre-primary education.	Lack of Anganwadi access.
		Absence of preschool facilities near settlements. Malnourishment affects cognitive development.
		No structured play or learning environment.
10.2	Empower and promote the social, economic and political inclusion of all.	Complete social invisibility. Lack of birth certificates or identity documents.
		Exclusion from government welfare schemes for children. Lack of voice in community affairs.
10.3	Ensure equal opportunity and reduce inequalities of outcome.	Institutional neglect.
		Caste and occupational stigma.
		No access to extra-curricular activities.
		Denied participation in school events.
		Daily experience of social rejection.

#### 3.3.1 Target 4.1: Free, equitable and quality primary and secondary education

The children of rag-pickers, even after the introduction of the Right to Education Act (2009), remain one of the most neglected and outcast groups in the education system. While there are schools near their settlements, there are numerous hurdles to even obtaining the most basic education. It was difficult for families engaged in rag-picking to understand the significance of education in attaining vertical mobility and enhancing one’s quality of life in the future. Rather, the immediate concern was day-to-day survival, which in many cases led to children as young as five joining the workforce either through accompanying parents to the rag-picking sites or assisting with household chores (Ghosh and Maity, 2025).

The majority of rag-pickers children do not attend school not because they cannot attend, but because they face exclusion based on identity, documentation and socio-economic factors. Many families did not own fundamental documents like birth certificates or Aadhar cards, which are often necessary for school admissions. In addition, the identity related stigma and discrimination coming from being part of a rag-pickers family deepened the sense of exclusion. Teachers from such schools were often reported to be indifferent or openly hostile towards such children. The lack of appropriate school uniforms, coupled with lack of books and mid-day meals that were not served on a regular basis exacerbated the difficulties.

*“His desire to attend school came to our notice and so we sought to enroll him to a local school. We went to the school, but their stance was that they could not offer admission unless we produced certain paperwork. The principal said he cannot be admitted without them. We went three times. After that, we gave up. Now he goes with his father to pick scrap” (Pooja).*

Out of the 12 participants (comprising of 23 children), only 07 children had been enrolled in formal schooling. Even for those enrolled, consistent attendance remained a significant challenge. Reasons included the burden of home or street work, lack of proper clothing and bullying by peers. All families were migrants from diverse linguistic backgrounds, which posed a challenge as well. The absence of inclusive classrooms and culturally relevant pedagogy further de-motivated learners from attending school (Banerjee and Duflo, 2011).

The lack of focused educational strategies like bridge courses and community education centers exacerbates the educational deficit. While initiatives like *Sarva Shiksha Abhiyan* and Government of India, Ministry of Education (2020) strive for inclusivity and the universalization of education, on-the-ground implementation is still fragmented and remains unattainable for the urban poor and migrants (Srivastava and Noronha, 2016). Mid-day meals, which serve as incentives for attendance, were insufficient or irregular. Many families reported that children were turned away for coming school without proper uniforms or shoes.

*“We sent our daughter to school but the teacher punished her for the dress she wore on that day. We are not affluent. We never sent her again. She now stays home to look after her younger brother while I go to my job” (Shakeela).*

Education is instrumental for addressing poverty in society both at the family level and communal level. In the case of children from rag-picking communities, there is a troubling gap between the policy of inclusive education and its implementation. It is not only a case of human rights violation, but an entire generation’s potential is being lost to the country (Ramachandran and Naorem, 2013).

#### 3.3.2 Target 4.2: Ensure that all girls and boys have access to quality early childhood development, care and pre-primary education

To facilitate effective development, children need to undergo early childhood development and health care services. Nevertheless, children of rag-pickers residing in urban slum areas are arguably the most neglected when it comes to receiving basic health services, nutrition and routine health checks. Their children often inhabit hovels located around garbage dumps, with vile squalor, polluted stagnant water and no drinking water conditions that are extremely damaging to their health and physical development (UNICEF, 2023).

Throughout the research, the majority of parents openly acknowledged that they had never scheduled routine health check-ups for their children. In most instances, vaccinations were either incomplete or entirely absent. Mothers as well as fathers were unaware of an immunization schedule or any supplemental nutritional aids provided by the government. This supports the argument of Arokiasamy et al. (2013), observing that children living in slums in India suffer from extreme health deprivation because of an absence of information, administrative obstacles and functional spatial inequity to health resources. One participant stated:

*“No, I do not think so. My children were never vaccinated. I do not know where or when such vaccinations are done. I had my children at home and never went to hospital for vaccinating my children” (Kumar).*

Another major concern is the widespread malnutrition of children, which severely hampers their cognitive skills and readiness for school. Data collected from the participants revealed that children who appeared to be underweight with stunted growth, they frequently appeared ill and lethargic. Their parents admitted to feeding their children stale food collected from the trash or inexpensive processed food that is low in nutritional value.

As reported in the NFHS-5 survey, children from urban poor households face a higher prevalence of wasting and stunting in comparison to children from higher income families (IIPS and MoHFW, 2021; Chakrabarty and Bharati, 2019). One participant stated that:

*“We eat what we get. Sometimes food from hotels, sometimes dry bread. Milk is rare. Fruits are for the rich. My child falls ill after every few days. We are not in a position to give nutritious food to our children because of poverty” (Iqbal).*

The prosthetic Anganwadi system, which forms the backbone of the India’s early childhood development programme, is inoperative or un-serviced in these informal slums. Parents reported that the closest Anganwadi center was either too far or would not admit the child due to lack of identification papers or due to their caste identity. This exclusion not only strips them of the right to supplementary nutrition and preschool education, but also deepens social discrimination. It has been mentioned that the persistent caste and class-based discrimination in Anganwadi centers acts as a fundamental obstacle to universal access (Dreze and Khera, 2017).

In addition, environmental concerns such as waste exposure, inhalation of noxious fumes and poor hygiene create an environment which is inhospitable for healthy development. Children walk and play in and around garbage, often barefoot, exposing them to infections and skin diseases. During fieldwork, the researchers observed several children were coughing persistently, a common symptom of respiratory infections aggravated by household air pollution and smoke exposure. One participant painfully narrated:

*“My son is always sick. Fever, cough, stomach pain are some of the health problems my child faces every week. But we cannot afford hospital or medicines, as the cost of medicines are very high which we being rag pickers cannot afford. We use home remedies or sometimes just wait” (Rukhsana).*

From the above narrative of the participant, it can assumed that there is a dire need to respond with a comprehensive and targeted health outreach for the health of children, particularly those living in informal settlements. Without health as a foundation, the aspiration of accessing quality early childhood care and education remains unattainable.

#### 3.3.3 Target 10.2: Empower and promote the social, economic and political inclusion of all

It has been observed that the children of rag-pickers face social exclusion, which renders them vulnerable and strips them of basic human rights. Nearly all participants of the study expressed that their social and political visibility had been eroded both as individuals and as a family unit, signaling the absence of governmental aid. Moreover, children are seldom registered at birth, which results in them lacking crucial identification papers such as Aadhaar and birth certificates. This absence of documentation greatly hampers their ability to access welfare schemes, education and healthcare (Jha, 2017).

According to the research, parents have repeatedly tried to access basic services like mid-day meals and pre-matric scholarships, all these things are hindered by the absence of proof of address or local guardian verification. This severely restricts the number of children eligible to receive social services. Moreover, participants reported that in the absence of such identity documents, they are often omitted from the lists generated for the ration distribution and education enrolment drives conducted by government agencies.

*“How can I bring proof when I do not have it?. I am an illiterate and have never heard or applied for these things. We are migrant rag pickers and here in Srinagar we are trying to earn for our children but lack of identity proof is creating a lot of problems for us. Nobody is ready to assist us here in Srinagar” (Salma).*

With regard to societal involvement, all participants stated a strong feeling of exclusion owing to their occupation. As the children of migrant rag-pickers are branded as *‘dirty’ or ‘untouchable,’* they are largely dissuaded from developing friendships or engaging in any community activities. This form of socio-economic exclusion is compounded by their caste background and the prejudice linked to the occupation of waste collecting, a phenomenon reported in numerous case studies of urban areas.

Ironically, the participants displayed a distinct lack of interest or concern. The majority seemed not to know about their voting rights or how to register to vote. They stated that no political party or community leader has reached out to them and they have not been to any public engagement or consultation meetings. Their exclusion from political engagement deepens their disempowerment and diminishes their influence in local governance (Sharma, 2020).

*“The construction of roads and drains is happening everywhere else, but nothing good is happening for us. We are living in tents and during rains we face many problems, as water enters into our tents. We are migrant workers and up to present day, no political leader or any other government official has come to verify about our conditions and problems” (P, 01, 03, 07, 08, and 12).*

On the basis of views of the participants, it can be said that the absence of documentation, combined with stigma stemming from occupation and bureaucratic indifference renders the children of rag-pickers during the socio-political exclusion. Their exclusion is not only actively sustained, but also goes unnoticed and without intervention from state mechanisms. Therefore, they become erasure within policy formulation and implementation and in turn, hinder the realization of SDG 10.2.

#### 3.3.4 Target 10.3: Ensure equal opportunity and reduce inequalities of outcome

In Jammu and Kashmir, the children of migrant rag-pickers face persistent and cumulative forms of inequality with regard to education and health services, as well as civic and social engagement. This long standing inequality is maintained by social stigma, occupational discrimination and lack of adequate and appropriate institutional response (Adhikari, 2016; Chatterjee, 2015). Even in the face of statutory safeguards and inclusive policies, the majority of children continue to be excluded from the active participation that is vital for individual development, social integration and citizenship.

Majority of the study participants shared that children within their households are routinely excluded from attending not only school, but also community events, social activities and extracurricular activities. School and community leaders actively discourage their participation in children’s clubs, sports events and cultural activities on grounds of lack of hygiene and appropriate clothing. Consequently, these children are deprived of education and in addition, the necessary character and confidence building (UNICEF, 2022).

*“When I inquired from my son’s teacher about participating in the school athletics day, she explained they cannot allow dirty children. My son stopped attending school after that” (Ramesh).*

Field interactions indicate that most children have never been enrolled in a formal school, even as many of them are first-generation learners. One participant, a father of three, shared:

*“Teachers ask our children to sit at the back and never encourage them to speak. They feel unwanted and stop going after a few days (Abdul).”*

One of the Participant revealed that, *“there is absence of preschools near the dumping grounds. “My child is four years old but has never held a book. There is no Anganwadi nearby and we cannot leave work to take them far” (Kamla).*

Mothers have reported that children of certain lower socioeconomic families are branded and socially ostracized in public spaces such as the market, clinics and parks as a result of rag-picking stains on their clothes and bodies. These forms of social exclusion perpetuate the already existing feelings of social and self-worth that children grapple with. Social exclusion on the basis of caste, occupation and neighborhood identity is not a new phenomenon (Iyer and Rao, 2024).

*“Inequitable structures of social welfare also systematically exclude children from rag-pickers families from government sponsored midday meals, scholarship opportunities, or grants for cultural activities because of lack of proper documentation or irregular school registration. They told me the midday meal can only be given when the child has a school ID or caste certificate. My daughter does not have either. So she just stays at home” (Nazira a mother of four children).*

The residential invisibility of such families has implications for civic engagement as well. None of the participant narrated being invited to the health camps at the ward level, parent-teacher meetings of the school, or polling booths within their vicinity. The residents within these areas are spatially invisible to municipal planners and are thus eliminated from urban planning considerations and municipal recognition, which spatially renders them publicly invisible.

The children of rag-pickers live with profound disadvantages which profoundly limit their access to opportunities for personal development, belonging and vital support systems. The goal of equal opportunity under SDG 10.3 continues to be elusive in the absence of deliberate strategies which remove barriers of lower caste stigma, identity documentation barriers and the absence of truly open pathways where participation is uninhibited.

## 4 Discussion

The findings of this study reveal a stark and painful situation where children of migrant rag-pickers in UT Jammu and Kashmir are still intentionally removed from the benefits of early childhood and pre-primary education, in clear violation of the national pledge in relation to Sustainable Development Goal 4.2. These children are spatially situated in marginalized settlements on the edges of urban society, living in grave physiologic and psychosocial deprivation and enduring constant exposure to traumatic stress and violence, living in dangerously neglected conditions near leaking garbage dumps. Economic instability has emerged as the primary barrier to accessing pre-primary education. Most families, because of their socio-economic conditions, push their children to rag-picking or domestic chores, sometimes as early as the age of three. This not only denies children the safety they require for healthy development, but also the critical chances to become developmentally ready for school (Ahmed, 2024). Furthermore, the seasonal or circular migratory patterns of rag-picker families mean that children are frequently displaced, hindering their ability to consistently attend Anganwadi centers or preschools.

Rag-picker children are also overlooked concerning policy execution. Programmes like Integrated Child Development Services (ICDS) and National Early Childhood Care and Education (ECCE) Policy have provisions, but for some reason, rag-pickers children are not considered under these schemes. The reasons are multi-layered, including but not limited to: lack of identification documents, informal housing not being recorded by the municipal authorities, and limited outreach of field officers to these at-risk areas. Anganwadi workers do not have the specialized training needed to work with children from marginalized and socially-ostracized families. Moreover, the centers are not only understaffed, but also underfunded, making them unable to meet the students’ needs (UNESCO, 2021). Cultural stigma and discrimination based on caste, alongside other forms of discrimination, deeply impact this issue. As a result of social exclusion, bullying and verbal abuse, many parents are hesitant to enroll their kids in Anganwadi centers. Children from families who are labeled as *“unclean”* due to their parent’s jobs often face bullying. These psychosocial factors create a system of exclusion and inequality (Teotia and Kumar, 2015).

The impact of education on breaking intergenerational poverty is well-studied. Still, in this context, SDG 4.2 cannot be met unless there are specific tailored strategies to address the unique challenges facing the children of rag pickers. This calls for a complete overhaul of how Early Childhood Care and Education (ECCE) is elevated and implemented, such as through mobile Anganwadis, community-based childcare networks, a culturally appropriate curriculum and collaboration with local NGOs and other civil society organizations active in urban informal settlements.

## 5 Conclusion

This study reveals that the children of migrant rag-pickers in Jammu and Kashmir are one of the most vulnerable populations that are systematically denied access to early and pre-primary education. Their experiences embody systemic abandonment that stems from structural poverty, administrative neglect and pervasive social discrimination. Many children were found to lack Aadhaar cards or a birth certificate, thereby restricting access to enrollment in school, vaccinations and child welfare schemes. The occupational stigma linked to their parents becomes a form of social surveillance that leads to institutional neglect and social ostracism for the children. The data collected in this study highlight how there is a need to create systems that are truly inclusive as well as consider the socio-educational challenges parents face with children of marginalized identities and ensure that these children are treated with dignity and compassion. The omission of these children from early educational provision is a grievous denial of their rights and a clear breach of the aspirations of SDG 4.2. The children of rag pickers also face various health problems because of the unhygienic conditions they are living in. What is essential is that governments, local organizations and NGOs shift to an inclusion and equity-based approach that is centered on the rights of the child. To guarantee access to early childhood education and healthy development as a universal entitlement and that early education is equitably accessible to all children, irrespective of their socio-economic background, policy structures must not adopt a blanket mobile and undocumented population paradigm.

## Data Availability

The raw data supporting the conclusions of this article will be made available by the authors, without undue reservation.
